# 45. Significantly Decreased Broad Spectrum Antimicrobial Use (Carbapenems And Fluoroquinolones) with Implementation of Antibiotic Stewardship Program (ASP) and Pharmacist Interventions

**DOI:** 10.1093/ofid/ofab466.247

**Published:** 2021-12-04

**Authors:** Ashlesha Kaushik, Sandeep Gupta, Corey Thieman, Michael Padomek

**Affiliations:** 1 UnityPoint Health and University of Iowa Carver College of Medicine, Sioux City, Iowa; 2 Pulmonary and Critical Care Medicine, Unity Point Health, Sioux City, Iowa; 3 Unity Point Health at St. Luke’s Regional Medical Center, Sioux City, Iowa

## Abstract

**Background:**

According to the WHO, carbapenems and fluoroquinolones (FQ) should be key targets for stewardship programs.

**Methods:**

A multifaceted antimicrobial stewardship program (ASP) was implemented in July 2018 at a 160-bed tertiary care center serving the tristate area of Iowa, South Dakota and Nebraska. Carbapenem and FQ use during pre-ASP intervention period (P1: 12/01/2016-6/30/2018) was compared with ASP-intervention period (P2: 07/01/2018-1/31/2020). ASP interventions included: stewardship educational pearls in monthly physician newsletters; educational posters in provider areas; suppression of carbapenem results on microbiology susceptibility reports; provider counseling for appropriate ordering; creating carbapenem alternative alert in order-entry software; removing FQ and carbapenems from order-sets where appropriate; default antibiotic stop dates changed to 7 days in EMR (Epic); adverse effects warning fired as an alert when ordering FQ. Pharmacist interventions: procalcitonin protocol allowing pharmacists to reorder follow-up procalcitonin and make recommendations to discontinue therapy where appropriate.

**Results:**

FQ use declined significantly from a mean of 133 days of therapy (DOT) per 1000 days to 46 DOT per 1000 patient days during P2 (p< 0.0001). Carbapenem use declined significantly from a mean of 65 DOT per 1000 patient days during P1 to 9 DOT per 1000 patient days in P2 (p< 0.001). All hospital units showed a significant decrease in use, with intensive care units (ICUs) noting 56% reduction (p< 0.00001) during P2 compared to P1. During P2, 55% of orders for carbapenems and FQ during P2 were found to be appropriate compared to 39% in P1 (p< 0.0001). Sensitivity profile for *Pseudomonas aeruginosa* improved from 86% carbapenem sensitivity during P1 to 89% in P2 and no *Carbapenem-Resistant Enterobacteriaceae* isolates were identified during the study period; FQ sensitivity remained stable at 81%. Cost savings of &757 per 1000 patient days were recognized in P2 as a result of reduced use.

**Conclusion:**

With ASP and pharmacist interventions, a significant decline in total utilization of carbapenem and FQ, considerable cost savings and an increase in proportion of appropriate use were observed.

**Disclosures:**

**All Authors**: No reported disclosures

